# Editorial: Autoinflammatory Keratinization Disease (AiKD)

**DOI:** 10.3389/fimmu.2020.01753

**Published:** 2020-08-05

**Authors:** Masashi Akiyama, Valerio De Vita, Kazumitsu Sugiura

**Affiliations:** ^1^Department of Dermatology, Nagoya University Graduate School of Medicine, Nagoya, Japan; ^2^Study Center of the Italian Group for Epidemiologic Research in Dermatology, Bergamo, Italy; ^3^Department of Dermatology, Fujita Health University School of Medicine, Toyoake, Japan

**Keywords:** autoinflammatory disease, hidradenitis suppurativa, KLICK syndrome, POMP, pityriasis rubra pilaris, porokeratosis, pustular psoriasis, skin

The hyper-activation of innate immunity caused by genetic factors sometimes results in skin inflammatory diseases with hyperkeratosis. Such cutaneous inflammatory disorders whose hyperkeratosis has genetic autoinflammatory pathomechanisms are termed “autoinflammatory keratinization diseases” (AiKDs) (1, 2). AiKDs also include diseases with mixed pathological mechanisms of autoinflammation and autoimmunity.

AiKDs involve significant genetic factors causing the hyper-activation of innate immunity (autoinflammation), primarily in the epidermis, and the upper dermis. The hyper-activation of innate immunity in the epidermis and the superficial dermis results in aberrantly up-regulated keratinization, leading to the additional inflammatory symptoms of AiKDs.

The shared clinical characteristics of AiKDs are hyperkeratotic inflammatory cutaneous lesions, although the clinical features of AiKDs vary and some AiKDs have unique clinical features. The majority of AiKD patients have recurrent and persistent cutaneous manifestations which are intractable against treatment with standard therapeutics for inflammatory keratinization disorders.

We believe that the idea of AiKDs provides us with clues to further understand the pathomechanisms behind various inflammatory keratinization diseases. The present Research Topic article aims to assess the debate over the pathomechanisms behind AiKDs, to propose avenues of investigation into therapeutic strategies for these diseases, and to deliver broad, divergent visions of how autoinflammation contributes to various cutaneous diseases.

The term “autoinflammatory diseases” (AiDs) was first introduced in 1999 when germline mutations in tumor necrosis factor receptor superfamily 1 (TNFRSF1) were reported to underlie tumor necrosis factor receptor-associated periodic syndrome (TRAPS) (also called familial Hibernian fever). Dramatic advances in the understanding of the mechanisms behind innate immunity have enabled us to designate inflammation caused by the genetic hyper-activation of innate immunity as “autoinflammation.” Monogenic disorders with systemic autoinflammatory symptoms are the principal AiDs. Kanazawa proposed that AiDs present various skin manifestations and that not a few AiDs were described as clinical entities originally from the skin symptoms. One example is cryopyrin-associated periodic fever syndrome (CAPS), which was originally called familial cold urticaria.

To elucidate the major skin symptoms of AiDs, Kanazawa introduced various AiDs that show skin manifestations, such as CAPS, Blau syndrome and pyogenic arthritis, acne and pyoderma gangrenosum (PAPA) syndrome. Information on the skin symptoms of AiDs shows the similarities and differences between AiDs and AiKDs, and lets us recognize that AiKDs are unique clinical entities distinct from AiDs.

Initially, AiKDs comprised pustular psoriasis and related disorders including generalized pustular psoriasis (GPP), impetigo herpetiformis and acrodermatitis continua due to mutations in *IL36RN* encoding the IL-36 receptor antagonist (IL-36Ra) (3), and GPP and palmoplantar pustular psoriasis due to *CARD14* variants, pityriasis rubra pilaris (PRP) caused by *CARD14* mutations/variants (4), and familial keratosis lichenoides chronica due to *NLRP1* mutations (5).

Akiyama described the rapid expansion of disorders categorized as AiKDs in recent years. Indeed, patients with hidradenitis suppurativa (HS), also called acne inversa, especially familial cases, have been proposed to have an AiKD (6). HS is a chronic, recurrent, progressive inflammatory disorder of the skin. Typical clinical features of HS are cystic abscess, comedones and sinus tracts in the axillae, groins and buttocks. Nomura summarized recent novel information on the genetic background and pathogenetic mechanisms underlying HS. From the advanced understanding of causative or disease-related genes, Nomura suggested that the pathomechanisms of HS are strongly associated with aberrantly activated keratinization and autoinflammation, and that HS should be recognized as an AiKD in the broad sense of the clinical entity. It is unknown whether the autoinflammatory events precede the hyperkeratotic changes in the hair follicle epithelia, or whether the hyperkeratosis precedes the autoinflammation in the pathogenesis of HS, but the occlusion of hair follicles is usually regarded as the primary event. Hyperkeratosis of the follicular epithelia and keratin plug formation are thought to play important roles in the primary stage of the autoinflammatory pathogenesis of HS cases with mutations in the γ-secretase genes *NCSTN, PSENEN* and *PSEN1* (7). However, variants in genes related to autoinflammation (e.g., *MEFV, NOD2, LPIN2, NLRP3, NLRP12, PSMB8, MVK, IL1RN, PSTPIP1*) have been reported to be associated with HS (8), and the variants probably cause autoinflammation preceding the follicular hyperkeratosis in the pathogenesis of HS. The fact that adalimumab, infliximab, anakinra, ustekinumab and other biologics are effective against HS may support the concept that HS should be categorized as an AiKD.

Furthermore, it may be possible to categorize keratosis linearis with ichthyosis congenita and sclerosing keratoderma (KLICK) syndrome as an AiKD (Takeichi and Akiyama). KLICK syndrome is a rare genodermatosis that clinically shows generalized ichthyosiform scaling, diffuse palmoplantar keratoderma with constriction rings on the fingers/toes, and hyperkeratotic plaques and papules distributed linearly on the arm folds and the wrists. Thickening of the spinous, granular and cornified cell layers in the epidermis and mild inflammatory cell infiltration are seen in affected skin. KLICK syndrome is due to a mutation in *POMP* which encodes proteasome maturation protein (POMP) (9). POMP plays an important role as a chaperone in proteasome maturation. Takeichi and Akiyama have suggested that proteasome insufficiency and disrupted proteasome assembly due to deficiency of POMP might result in both the hyperkeratosis and the autoinflammation seen in the skin in KLICK syndrome. Thus, Takeichi and Akiyama have proposed that KLICK syndrome due to the deletion mutation in the 5′ untranslated region of *POMP* be categorized as a proteasome-associated AiKD. In fact, mutations in *POMP* are also reported to cause an AiD: proteasome-associated autoinflammatory syndrome-2.

In addition to the diseases discussed above, porokeratosis with mutations in four mevalonate pathway genes (*MVK, MVD, PMVK* and *FDPS*) is also suggested to be an AiKD (10).

Akiyama expects that increasing numbers of inflammatory keratinization diseases will be included in AiKDs in the future as the pathogenic mechanisms of inflammatory keratinization diseases in the skin are successively elucidated. AiKD as a novel clinical entity has provided us with a novel concept of inflammatory keratinization diseases. In addition, advances in the understanding of the aberrant hyper-activation of innate immunity in the skin as pathomechanisms behind AiKDs could lead us to innovations in more effective, targeted, causal treatments for various AiKDs.

## Author Contributions

All authors listed have made a substantial, direct and intellectual contribution to the work, and approved it for publication.

## Conflict of Interest

The authors declare that the research was conducted in the absence of any commercial or financial relationships that could be construed as a potential conflict of interest.
